# DNA image cytometric analysis of bronchial washings as an adjunct for the detection of lung cancer in a clinical setting

**DOI:** 10.1002/cam4.4574

**Published:** 2022-02-11

**Authors:** Yan Hu, Qing Yu, Cuiyan Guo, Guangfa Wang

**Affiliations:** ^1^ Department of Respiratory and Critical Care Medicine Peking University First Hospital Beijing China

**Keywords:** aneuploidy, bronchial washing, bronchoscopy, DNA image cytometry, lung cancer

## Abstract

**Background:**

DNA aneuploidy has a potential to become an adjunct to conventional cytology for diagnosis of lung cancer, but its value in bronchial washings has not been well evaluated.

**Methods:**

We conducted a retrospective study on patients who underwent bronchoscopy and the bronchial washings were submitted for both cytology and DNA image cytometry (DNA‐ICM) examination. The sensitivity and specificity of two methods and both in combination were compared. Analysis of clinical subgroups and DNA histogram were also performed.

**Results:**

The study included 626 patients (326 patients with lung cancer and 300 patients with benign lung diseases). The sensitivity of cytology, DNA‐ICM, and combination test for lung cancer were 53.3%, 62.3%, and 75.8%, respectively, and the sensitivity of DNA‐ICM and combination test were superior to that of cytology (*p* < 0.05). A modest reduction of specificity was found in DNA‐ICM compared with cytology (91.3% vs. 98.3%, *p* < 0.05). Subgroup analysis showed there was no significant difference in sensitivity of DNA‐ICM between the visible tumor group and the invisible tumor group (66.5% vs. 56.9%, χ^2^ = 3.114, *p* = 0.078). Among 101 patients with invisible endobronchial tumor, the positive rates for DNA‐ICM of washing, cytology of washing, brushing and biopsy were 62.4%, 41.6%, 40.6%, and 45.5%, respectively. DNA‐ICM in combination with the basic bronchoscopy techniques could increase the sensitivity from 67.3% to 87.1% (*p* = 0.000). The DNA histogram analysis showed 25.3% washing samples of lung cancer were diploid pattern, 49.4% were scattered aneuploid cells pattern, and 25.3% were aneuploid peaks pattern. Small cell lung cancer had the highest proportion of aneuploid peaks pattern (*p* < 0.05).

**Conclusions:**

DNA‐ICM could be used as an adjunct for the detection of lung cancer. The combination of DNA‐ICM and basic bronchoscopy techniques could significantly increase the sensitivity, especially for the patients suspected of peripheral lung cancer, and contribute to select subjects for advanced bronchoscopy.

## Graphical abstract

This study showed that computer‐assisted DNA image cytometry (DNA‐ICM) analysis of bronchial washings was a simple and useful adjunct for the detection of lung cancer. The combination of DNA‐ICM and cytology could significantly improve the sensitivity with an acceptably modest reduction in specificity.

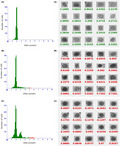

## 1 INTRODUCTION

Lung cancer is the leading cause of cancer‐related death in the world.^1^ It is important to establish a timely and accurate method for diagnosing lung cancer in the clinical setting. Flexible bronchoscopy has been widely used in the patients with suspected lung cancer. Bronchial washing is one of the safe and cost‐effective universal diagnostic techniques for all patients undergoing bronchoscopy.^2^ However, as the cytological evaluation of bronchial washing is influenced by many sampling issues, such as few malignant cells in specimen, obscuration by blood, squamous contamination, and cellular crushing, the possibility of false‐negative results may not be excluded.^3^, ^4^, ^5^ Moreover, the poor differentiation of cancer cells sometimes leaves an equivocal or inconclusive result even in the hands of experienced observers.^6^

Nuclear DNA content increases with degree of cellular atypia up from mildly dysplastic changes to invasive cancer. The emergence of DNA content abnormality is often a critical early event during carcinogenesis.^7^ By cytometric evaluation of nuclear DNA content after stoichiometric staining of DNA, a DNA stem line in an abnormal histogram position is often termed a “DNA aneuploidy,” which has been thought to be a reliable marker of cancer.^8^, ^9^ Computer‐assisted DNA image cytometry (DNA‐ICM) is a sensitive method for the quantitative analysis of the nuclear structure and DNA content of individual cells. Several studies have demonstrated the advantage of DNA‐ICM in diagnosing various malignant cancer types with objectivity, convenience, and a high positive rate.^7^, ^9^, ^10^, ^11^, ^12^, ^13^

However, the DNA aneuploidy is also present when cells are affected by infection, inflammation, or injury.^6^, ^14^, ^15^ Therefore, the specificity of DNA‐ICM for lung cancer will be dependent on the characteristics of the cohort studied. When sputum DNA‐ICM was applied to detect lung cancer in high‐risk patients and controls, a high specificity and an increased sensitivity compared to conventional cytology were reported.^16^, ^17^, ^18^ But in a clinical setting mixed with a variety of nonmalignant pulmonary diseases, the specificity of DNA‐ICM for diagnosing lung cancer is obviously decreased.^14^

Bronchoscopy is usually performed in patients with abnormalities on CT scans. The final diagnosis would be various. The clinical value of DNA‐ICM analysis of bronchial washings in a complex real‐world setting has not been well evaluated. The aim of this study was to compare the diagnostic accuracy of cytology, DNA‐ICM, and both in combination in detecting lung cancer cells in bronchial washings.

## 2 MATERIALS AND METHODS

### 2.1 Patient samples

The study was approved by the Ethics Committee of Peking University First Hospital. Consecutive bronchial washing samples were collected from patients who underwent bronchoscopy at Peking University First Hospital from September 2016 to December 2019. The clinical data, laboratory test results, radiographic findings, and pathological results from bronchoscopy, convex probe and radial probe endobronchial ultrasound (EBUS), percutaneous lung biopsy, and surgical operation were also reviewed. The histological results were the golden standard for the diagnosis of lung cancer. It was reported that DNA‐ICM was able to diagnose malignant tumors 1–15 months before histology can.^19^, ^20^ Therefore, the outcomes of the participants who were not diagnosed as lung cancer by histological examination were collected at 18 months after the bronchoscopy to discover the potential malignancy. The diagnosis of benign diseases was based on the comprehensive evidence of clinical manifestation, imaging findings, laboratory results, pathological features, and treatment response.

Inclusion criteria were (i) subjects with a definite diagnosis, (ii) cytologic analysis by two methods, liquid‐based cytology and automatic DNA‐ICM separately for the same bronchial washing sample, and (iii) at least 500 nuclei of epithelial cells contained in each specimen.^21^ Exclusion criteria were (i) malignant diseases with a non‐pulmonary origin (e.g., mesotheliomas, metastasis, and lymphoma), (ii) subjects who had received radiotherapy, chemotherapy, or cytotoxic drugs therapy within 1 year before bronchoscopy, and (iii) subjects who were lost to follow‐up.

### 2.2 Cytology diagnosis

The washing samples were submitted for liquid‐based cytologic examination at the Pathology Department of Peking University First Hospital. The specimens were centrifuged for 5 min at 1200 rpm after dithiothreitol treatment, and the supernatants were discarded. The sediment was placed into a vial of ThinPre ReservCyt solution (Hologic) and left to stand for 15 min. The vial was run on an automated ThinPrep 2000 processor (Hologic), yield a liquid‐based slide. The cells were fixed with 95% ethyl alcohol for 10 min and then stained with hematoxylin and eosin. All slides were interpreted by two cytopathologists, who were blinded to the patients' clinical information and the DNA‐ICM results. Cytological results that indicate malignant cells and suspicious for malignant cells were classified as positive.

### 2.3 Automated DNA‐ICM

The specimens underwent centrifugation at 2000 rpm for 5 min at room temperature and the supernatant was discarded. Then 50% ethyl alcohol was added to resuspend the cells. The cells were mounted on microscope slides by cytocentrifugation (1300 rpm for 5 min) forming a uniform thin deposition layer. All slides were stained with the DNA specific and stoichiometric (Feulgen–Thionin) stain according to the method described by Feulgen.^22^ A fully automated, high‐resolution image cytometer (DNA Ploidy Analysis System from Landing Medical Hi‐Tech Co., Ltd.) was used to measure nuclei characteristics and DNA distribution in all nucleated cells present in the sample, according to previously described technical details.^11^ The resulting DNA ploidy value is expressed as a “c” value for chromosome. A DNA ploidy value of 2c indicates a normal diploid cell and 4c a tetraploid cell. Aneuploid is defined as polyploidy with a DNA content of more than 5c, which is a cutoff used by most authors. Most studies suggest that the threshold for assuming malignancy is the existence of more than three nuclei with DNA content greater than 5c.^11^, ^23^, ^24^, ^25^ Schramm et al.^6^ favor the identification of abnormal position of any DNA stemline and/or at least one cell with a DNA content >9c as the diagnostic criteria for lung cancer, since octaploid cells rarely occur in noncancerous epithelium of inflamedlungs.^15^ Therefore, the positive indicators for DNA‐ICM in our study were three or more cells >5c and/or occurrence of at least one cell >9c. All technical instruments, all software used, and guidelines for diagnostic interpretation and quality assurance met the standard requirements of the consensus reports of the European Society for Analytical Cellular Pathology.^26^

### 2.4 Statistical analysis

Sensitivity, specificity, positive predictive value, and negative predictive value were calculated for cytological diagnosis, DNA‐ICM, and the combined test. The statistical difference in the sensitivity and specificity for diagnosis of lung cancer between cytology and DNA‐ICM was determined using McNemar's test. The positive rates of cytology and DNA‐ICM in different subgroups according to pathological types and bronchoscopic findings were assessed using the chi‐square test. Based on DNA histogram profiles, the proportion of different DNA patterns in each pathological subgroup was compared by the chi‐square test. All analyses were performed using SPSS 20.0 software (IBM Corp.,). Statistical significance was defined as a two‐sided *p* < 0.05.

## 3 RESULTS

### 3.1 Patient characteristics

**FIGURE 1:**
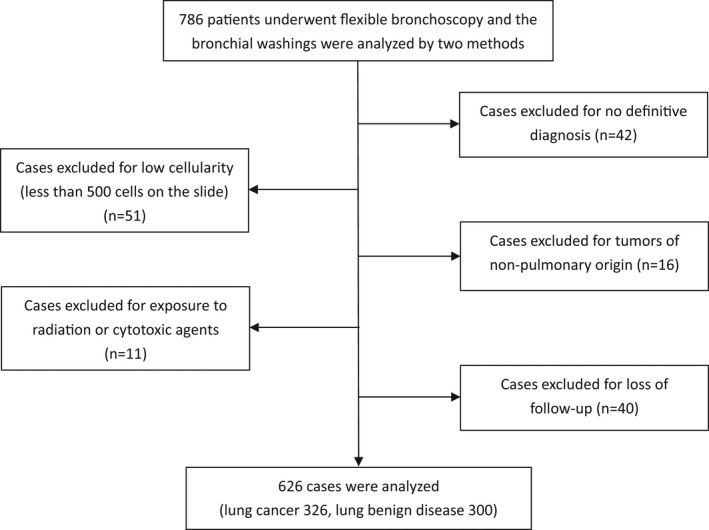
The flow chart of patient selection for the study

A total of 626 patients were enrolled (Figure 1). The median age of the patients was 63 (range 20–84) years, and there were 397 (63.4%) males. This study included 326 patients with lung cancer and 300 patients with lung benign diseases (Table 1). The pathological examination showed 119 (36.5%) cases of adenocarcinomas (ADC), 90 (27.6%) cases of squamous cell carcinomas (SQC), 68 (20.9%) cases of small cell carcinomas (SCC), and 49 (15.0%) cases of other non‐small cell carcinomas (NSCC). In our study, the lung benign diseases included pulmonary infection (31.0%), nonspecific inflammation (27.7%), benign nodule (12.0%), interstitial lung disease (11.3%), sarcoidosis (7.0%), benign bronchial stenosis (7.0%), and mediastinal fibrosis (4.0%).

**TABLE 1:** The final diagnosis of all patients

Lung cancers	326
Adenocarcinoma	119 (36.5%)
Squamous cell carcinoma	90 (27.6%)
Small cell carcinoma	68 (20.9%)
Other non‐small cell carcinoma	49 (15.0%)
Benign diseases	300
Pulmonary infection	93 (31.0%)
Pneumonia	42
Pulmonary abscess	15
Fungal infection	7
Tuberculosis	29
Nonspecific inflammation	83 (27.7%)
Benign nodule	36 (12.0%)
Interstitial lung disease	34 (11.3%)
Sarcoidosis	21 (7.0%)
Benign bronchial stenosis	21 (7.0%)
Mediastinal fibrosis	12 (4.0%)

### 3.2 Cytology, DNA‐ICM, and the combined tests for diagnosis of lung cancer

The sensitivity of DNA‐ICM and cytology for lung cancer was 62.3% and 53.3%, respectively, and that of DNA‐ICM was superior according to McNemar's test (*p* = 0.015). The combination of cytology and DNA‐ICM significantly improved the sensitivity up to 75.8% (*p* = 0.000). A modest reduction of specificity was found in DNA‐ICM compared with cytology (91.3% vs. 98.3%, *p* = 0.000). The sensitivity, specificity, positive predictive value, and negative predictive value of cytology, DNA‐ICM and combined tests are listed in Table 2.

**TABLE 2:** Diagnostic accuracy of cytology, DNA‐ICM and combination tests

	Cytology	DNA‐ICM	Combination
Sensitivity (%) [95% CI]	53.3 [47.7–58.8]	62.3 [56.7–67.5]	75.8 [70.7–80.2]
Specificity (%) [95% CI]	98.3 [95.9–99.4]	91.3 [87.4–94.2]	90.0 [85.9–93.0]
PPV (%) [95% CI]	97.1 [93.1–98.9]	88.6 [83.6–92.3]	89.2 [84.8–92.5]
NPV (%) [95% CI]	66.4 [61.8–70.8]	69.0 [64.2–73.5]	77.4 [72.5–81.6]

Abbreviations: DNA‐ICM, DNA image cytometry; NPV, negative predictive value; PPV, positive predictive value.

Neither cytology nor DNA‐ICM exhibited significant difference in the positive rates among different histological types of lung cancer (cytology, χ^2^ = 6.710 *p* = 0.082; DNA –ICM, χ^2^ = 2.855 *p* = 0.415). Based on the manifestation of white light bronchoscope, the patients with lung cancer were divided into two subgroups, visible endobronchial tumor group and invisible endobronchial tumor group. The “visible tumor” was defined as an endobronchial mass protruding into the lumen of bronchus. The “invisible tumor” was defined as normal bronchoscopic findings, mild mucosal swelling, or external compression with normal mucosa. For DNA‐ICM, there was no significant difference in the positive rates between the two subgroups (66.5% vs. 56.9%, χ^2^ = 3.114, *p* = 0.078). However, the positive rate of cytology in invisible tumor group was significantly lower than that in visible tumor group (39.6% vs. 64.8%, χ^2^ = 20.792, *p* = 0.000). Therefore, for the invisible tumor group, DNA‐ICM could increase the positive rate from 39.6% of cytology up to 56.9% (*p* = 0.001). But there was no significant difference in positive rates between DNA‐ICM and cytology in the visible tumor group according to McNemar's test (*p* = 1.0). The results of subgroup analysis based on histological types and bronchoscopic findings are shown in Table 3.

**TABLE 3:** The positive rate of cytology and DNA‐ICM in bronchial washings of patients with lung cancer

Subgroups	No. (%) *n* = 326	Positive rate of cytology		Positive rate of DNA‐ICM	
		No. (%)	*p* value	No. (%)	*p* value
Histological types					
ADC	119 (36.5)	57 (47.9)	0.082	70 (58.8)	0.415
SQC	90 (27.6)	48 (53.3)		62 (68.9)	
NSCC	49 (15.0)	23 (46.9)		28 (57.1)	
SCC	68 (20.9)	45 (66.2)		43 (63.2)	
Bronchoscopic findings					
Visible tumor	182 (55.8)	118 (64.8)	0.000	121 (66.5)	0.078
Invisible tumor	144 (44.2)	57 (39.6)		82 (56.9)	

Abbreviations: ADC, adenocarcinoma; NSCC, non‐small cell carcinoma; SCC, small cell carcinoma; SQC, squamous cell carcinoma.

**FIGURE 2:**
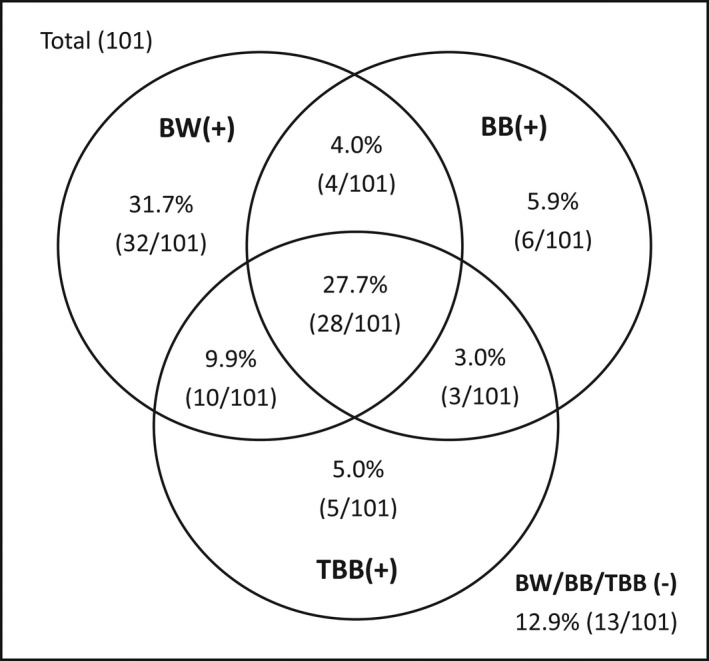
The positive rates of different techniques of bronchoscopy in the lung cancer patients with invisible endobronchial tumor. BW, bronchial washing; BB, bronchial brushing; TBB, transbronchial biopsy. +, positive results; − negative results

Since the DNA‐ICM showed advantages in sensitivity of the invisible tumor group, we performed further analysis in this subgroup. There were 101 lung cancer patients with invisible endobronchial tumor who underwent bronchial washing (cytologic analysis by both DNA‐ICM and cytology), brushing, and transbronchial biopsy in the same bronchoscopy session. The positive rates for DNA‐ICM of washing, cytology of washing, brushing, and biopsy were 62.4%, 41.6%, 40.6%, and 45.5%, respectively. The combination of DNA‐ICM and the basic bronchoscopy techniques could increase the total positive rate from 67.3% up to 87.1% (*p* = 0.000). The positive rates of different diagnostic procedures are shown in Figure 2.

In 300 patients who had benign diseases, DNA‐ICM produced 26 false positives, while cytology had 4 false positives. The final diagnoses of the patients who got false‐positive results of DNA‐ICM were pneumonia (8/26), benign nodule (4/26), pulmonary abscess (3/26), tuberculosis (3/26), benign bronchial stenosis (3/26), mediastinal fibrosis (2/26), nonspecific inflammation (2/26), and interstitial lung disease (1/26). In the patients diagnosed with benign diseases, 9.3% (28/300) of them had purulent secretions in the airway. And 39.3% of those (11/28) who presented with purulent airway secretions had false‐positive results of DNA‐ICM, accounting for 42.3% (11/26) of total false‐positive cases.

### 3.3 DNA histogram profiles of lung cancer

**FIGURE 3:**
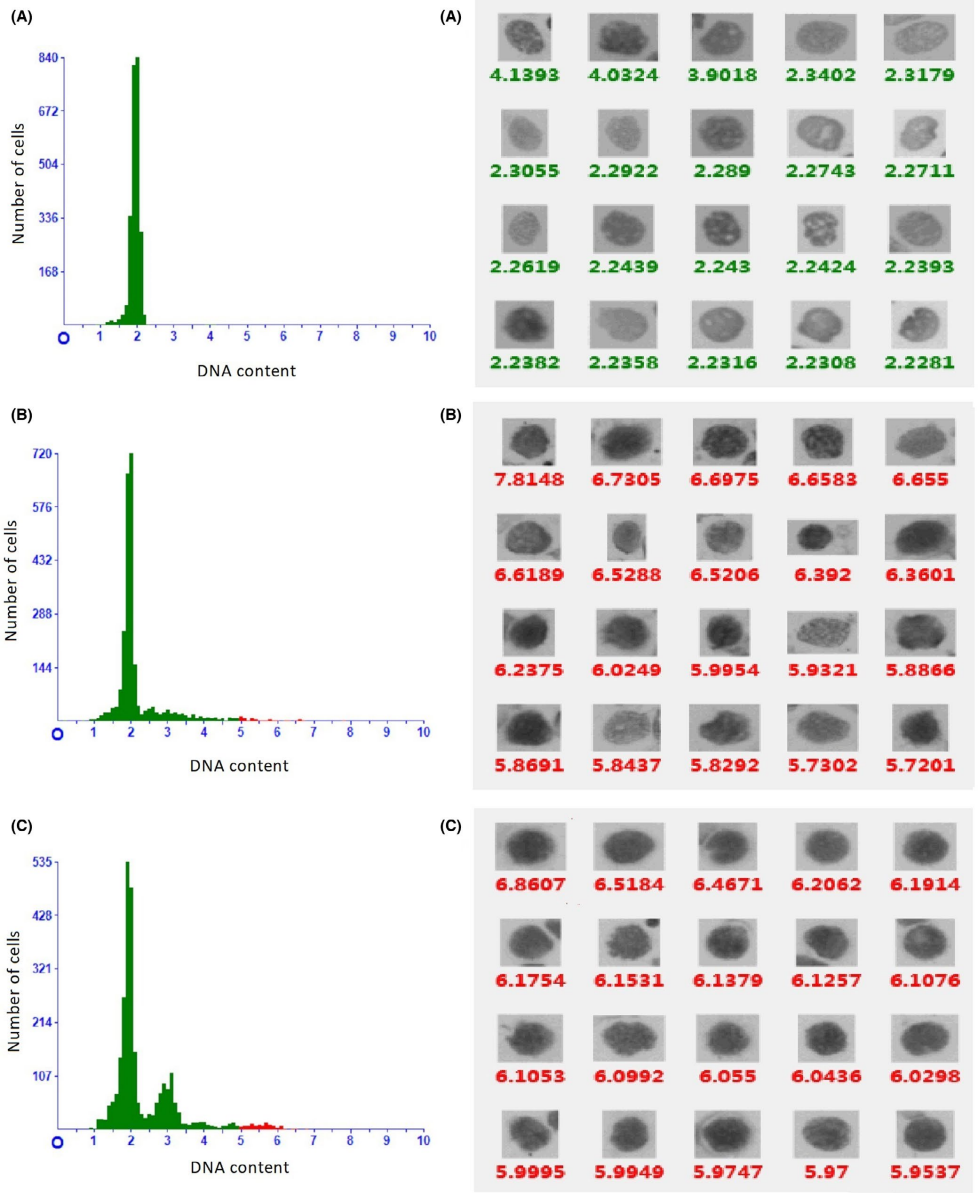
The results of automated DNA image cytometry. The left images illustrate DNA histogram, that is, DNA content (x‐axis) is plotted against number of cells (y‐axis). The right images illustrate the DNA content of every nucleus of different cells in the specimens (arranged from large to small according to DNA content). A, diploid pattern (a diploid peak on DNA histogram); B scattered aneuploid cells pattern (a diploid peak accompanied by several >5c aneuploid cells); C multiple aneuploid peaks pattern (DNA histogram revealing aneuploid DNA stemlines at 3c, 4.8c and 5.6c). The red spots represent >5c cells

DNA histogram analysis was performed in the washing specimens in which the lung cancer cells were detected by cytology (*n* = 174). DNA histograms were classified into three patterns. It was classified as diploid pattern if there was only one peak (which was 2c) during the G_0_ or G_1_ phase, if the number of 4c nuclei during the peak of the G_2_ phase did not exceed 10 % of total, or if the number of nuclei with a DNA content of more than 5c did not exceed two. It was defined as scattered aneuploid cells pattern if there was a diploid peak accompanied with three or more scatted >5c cells or occurrence of cells >9c. It was defined as aneuploid peaks pattern if there were one or multiple aneuploid peaks shown on DNA histograms. The DNA histograms of corresponding patterns are shown in Figure 3. Among 174 washing specimens of lung cancer, 44 (25.3%) were classified as diploid pattern, 86 (49.4%) exhibited scattered aneuploid cells pattern, and 44 (25.3%) showed as aneuploid peaks pattern (Table 4). There was no significant difference in the percentage of diploid pattern among different histological subgroups. However, SCLC subgroup had a significantly higher proportion of aneuploid peaks pattern and a lower proportion of scattered aneuploid cells pattern compared with other non‐small cell lung cancer groups (ADC, SQC, and NSCLC groups) (*p* < 0.05).

**TABLE 4:** DNA histogram analysis based on histological types

DNA histogram patterns	No. (%)	ADC	SQC	NSCLC	SCLC
		No. (%)	No. (%)	No. (%)	No. (%)
Diploidy	44 (25.3)	17 (29.8)	9 (18.8)	5 (21.7)	13 (28.3)
Scattered >5c cells	86 (49.4)	29 (50.9)	33 (68.8)	13 (56.5)	11 (23.9)*
Aneuploid peaks	44 (25.3)	11 (19.3)	6 (12.5)	5 (21.7)	22 (47.8)*
Total	174	57	48	23	46

* Adjust (Bonferroni method) *p* < 0.05 compared with other histological subgroups respectively. ADC, adenocarcinoma; NSCC, non‐small cell carcinoma; SCC, small cell carcinoma; SQC, squamous cell carcinoma.

## 4 DISCUSSION

In this study, the sensitivity and specificity of DNA‐ICM in detecting lung cancer cells in bronchial washings were 62.3% and 91.30%, respectively. The combination of DNA‐ICM and cytology had a significant improvement of sensitivity (75.8% vs. 53.3%, *p* = 0.000) and a modest reduction of specificity (90.0% vs. 98.3%, *p* = 0.000) compared with conventional cytology. In particular, in patients with invisible endobronchial tumor, DNA‐ICM significantly increased the positive rate from 39.6% of cytology up to 56.9%. There was no significant difference in the positive rates of DNA‐ICM among different histological types of lung cancer.

As a proven cancer biomarker, DNA aneuploidy has a potential to become an adjunct to conventional cytology for diagnosis of lung cancer. Both flow cytometry (FCM) and ICM can be used for quantitative analysis of cellular DNA. It is reported that DNA ploidy analysis by ICM is more accurate than by FCM,^27^, ^28^ and the specimen preparation for DNA‐ICM is simpler. The DNA‐ICM can automatically identify changes of DNA content in single cells by scanning the specimen slide with a fully automatic digital microscope. DNA‐ICM has been used to detect lung cancer cells in sputum,^13^ pleural effusion,^24^ bronchial alveolar lavage (BALF) or washing,^29^ and bronchial brush.^30^ Using a PubMed search, we reviewed the previously reported results of DNA‐ICM analysis of BALF or washing for diagnosis of lung cancer. Four relevant studies were found.^6^, ^23^, ^29^, ^30^ From the literature review, the sensitivity of DNA‐ICM was 69.05%~90%, and the specificity was 80.28%~100% (Table 5). To our knowledge, this is the largest research on the application of DNA‐ICM analysis of bronchial washings in lung cancer diagnosis.

**TABLE 5:** Sensitivity and specificity of DNA‐ICM for lung cancer based on a literature review

First author	Year	No. of patients	Specimen type	Sensitivity	Specificity
Marek W^29^	1999	142 patients with suspected lung cancer and 50 controls (COPD, asthma)	BW	90%	84%
Schramm M^6^	2011	179 patients with suspected lung cancer	BW/BB/TBNA	79.0%	98.2%
Tao W^30^	2016	216 lung cancer patients, 209 benign lung diseases patients	BALF/BB	69.2%	100%
Shi A^23^	2018	386 suspected lung cancer patients	BALF	69.05%	80.28%

Abbreviations: BALF, bronchial alveolar lavage fluid; BB, bronchial brushing; BW, bronchial washing; TBNA, transbronchial needle aspiration.

Subgroup analysis suggested that the diagnostic superiority of DNA‐ICM existed in patients with invisible endobronchial tumor. Nodit L et al. performed a root cause analysis of false‐negative bronchial brushing and washing specimen errors in lung cancer cases.^5^ In 59% (19/32) false‐negative cases, tumor was not present on the cytologic specimen, although the tumor was submucosal or tumor broached the mucosal surface on the surgical biopsy tissue. This problem might be more common in the cases with invisible endobronchial tumor. Nevertheless, DNA‐ICM is a more sensitive method than conventional cytology, since atypical and malignant cells prior to morphological changes can be found by measuring nuclear DNA content.^13^ Moreover, the automatic computer‐assisted cytometry makes it sensitive for detection of rare aneuploid cells. In some lung cancer cases, it was reported that the bronchial washings were positive detected by DNA‐ICM while negative by conventional cytology.^23^ We speculate that the advantage of DNA‐ICM in sensitivity offsets the disadvantage of sampling issues in patients with invisible endobronchial tumor, hence there was no significant difference in the positive rate of DNA‐ICM between the visible tumor group and the invisible tumor group.

On the other hand, certain malignant tumors exhibit diploid DNA, leading to false‐negative results of DNA‐ICM. Barlogie et al. reported that aneuploidy was found in leukemia (23% among 793 patients), in lymphoma (53% among 360 patients), and in myeloma (76% among 177 patients), as well as in solid tumors (75% among 3611 patients), for an overall incidence of 67% in 4941 patients.^31^ A meta‐analysis of 35 related studies reported the prevalence of aneuploidy in NSCLC at 65% (95% CI: 64%–67%).^32^ Our study showed that 74.7% of lung cancer presented with aneuploidy, and 25.3% was diploidy. The highest proportion of diploidy was 29.8% in ADC subgroup, and the lowest was 18.8% in SQC subgroup. But the difference was not statistically significant. We speculate that the false negative of DNA‐ICM due to existence of diploidy in lung cancer offset its superiority over cytology in patients with visible endobronchial tumor, so there was no significant difference in the positive rate of the visible tumor group between DNA‐ICM and cytology.

Therefore, DNA‐ICM could be used as a diagnostic adjunct to conventional cytology, especially for the patients suspected of peripheral lung cancer. Currently, advanced bronchoscopy techniques, such as electromagnetic navigation bronchoscopy, radial probe EBUS, ultrathin bronchoscopy, and virtual bronchoscopy, have facilitated the diagnosis of peripheral lung cancer. But these advanced bronchoscopy techniques are expensive and not available in many institutions. The combination of DNA‐ICM and basic techniques of bronchoscopy could significantly increase the sensitivity in detecting lung cancer, and contribute to select subjects for advanced bronchoscopy.

Aneuploidy cells peaks, especially multi‐peaks, displayed in the histogram as continuous peaks with different heights like Manhattan skyline, probably indicate a high genotypic instability. Although several studies have reported that DNA aneuploid in lung cancer is associated with a poor outcome,^32^, ^33^ few studies focus on the DNA ploidy profiles in different histological types of lung cancer. Our study showed that the proportion of aneuploid peaks pattern in SCLC was significantly higher than that in NSCLC subgroups, in consistent with the more aggressive biological characteristics of SCLC. However, Du Y et al. found no significant differences in the aneuploid cell peaks among SCLC, ADC, and SQC by detecting bronchial brush specimens with DNA‐ICM.^34^ Further studies are needed to demonstrate whether the DNA ploidy profiles have prognostic and predictive significance in lung cancer.

It is reported that the cells with DNA content of more than 5c could possibly be found in some benign lung disease.^14^, ^15^ One of mechanisms could be cellular damage induced by oxygen radicals or inflammation, which is a common reaction preceding or present in a variety of diseases. In our study, 8.7% (26/300) patients with benign lung diseases had false positive results of DNA‐ICM. It is worthy to note that 42.3% of patients with false positive of DNA‐ICM presented with airway purulent secretions. No evidence of lung cancer was found in these patients during subsequent follow‐up. Therefore, we speculate that the suppurative inflammation might lead to aneuploidy. Hamada S et al. reported that inflamed bronchial epithelial cells could contain a DNA content up to 8c.^15^However, aneuploid cells with the maximum DNA content of 17c were found in a patient with lung abscess in our study. The false positive in DNA‐ICM makes its specificity significantly lower than that of cytology, so the results of DNA‐ICM should be interpreted with caution. Since nearly half of the false‐positive cases existed in the patients with suppurative pulmonary infection, if DNA‐ICM is used in the patients without clinical manifestations of pulmonary infection, the specificity of DNA‐ICM is presumed to increase.

Our study has some limitations. First, this is a retrospective study. The patients lost to follow‐up and the specimens with low cellularity were ruled out of this study, so the results might be influenced by possible selection bias. Second, the patients who had false‐positive results of DNA‐ICM did not undergo a second bronchoscopy after the remission of pulmonary infectious diseases, so the possibility of bronchial precancerous lesions or carcinoma in situ could not be ruled out. Therefore, a prospective study involving follow‐up with CT and bronchoscopy is needed.

In conclusion, the combination of DNA‐ICM and cytological evaluation of bronchial washings could improve the sensitivity for detecting lung cancer, especially for the patients with invisible endobronchial tumor. It should be noted that some benign lung diseases, especially suppurative infection, could lead to false positive of DNA‐ICM, and meanwhile the diploid lung cancer could result in false negative of DNA‐ICM. Therefore, we recommend combination of DNA‐ICM and other bronchoscopic techniques in the patients suspected of lung cancer, especially for suspected peripheral lung cancer.

## CONFLICT OF INTEREST

The authors declare no conflict of interest.

## AUTHOR CONTRIBUTIONS

Yan Hu was involved in conceptualization, methodology, investigation, data analysis, and writing (original draft). Qing Y was involved in methodology, investigation, and data collection and analysis. Cuiyan Guo was involved in investigation, methodology, and resources. Guangfa Wang was involved in conceptualization, methodology, supervision, and writing (review and editing).

## Data Availability

Data sharing is not applicable to this article as no new data were created or analyzed in this study.
